# Mitigating Bovine Mastitis and Raw Milk Pathogen Risks: Inhibition of *Staphylococcus xylosus* by Mediterranean Plants’ Essential Oil

**DOI:** 10.3390/vetsci12070659

**Published:** 2025-07-11

**Authors:** Rosario De Fazio, Giacomo Di Giacinto, Paola Roncada, Domenico Britti, Rosangela Odore, Paola Badino, Cristian Piras

**Affiliations:** 1 Department of Health Sciences, Magna Græcia University of Catanzaro, 88100 Catanzaro, Italy; rosario.defazio@studenti.unicz.it (R.D.F.); roncada@unicz.it (P.R.); britti@unicz.it (D.B.); 2 Department of Veterinary Sciences, University of Turin, Largo Braccini 2, Grugliasco, 10095 Torino, Italy; giacomo.digiacinto@unito.it (G.D.G.); rosangela.odore@unito.it (R.O.); paola.badino@unito.it (P.B.); 3 Interdepartmental Center Veterinary Service for Human and Animal Health, University “Magna Graecia” of Catanzaro, CISVetSUA, 88100 Catanzaro, Italy

**Keywords:** raw milk, food safety, *Staphylococcus xylosus*, bovine mastitis, antimicrobial resistance, essential oils, antimicrobial activity, MALDI biotyper

## Abstract

Milk is frequently susceptible to contamination by potential pathogens, posing risks to both food safety and public health. Cheesemaking often relies on raw milk, where microbial communities—including *Staphylococcus xylosus*—can play a dual role: (i) contributing to fermentation and (ii) acting as opportunistic pathogens that can be often present in subclinical mastitis and be subjected to carry over in dairy products. In this study, *Staphylococcus xylosus* was isolated from raw bovine milk (preclinical mastitis) and identified via matrix-assisted laser desorption/ionization time-of-flight mass spectrometry (Biotyper scores: 1.87–2.19). Its susceptibility to erythromycin and to an essential oil blend composed of *Myrtus communis*, *Salvia officinalis*, and *Cistus ladanifer* was evaluated. The essential oil blend produced inhibition zones ranging from 9 mm to 13.3 mm, indicating moderate antibacterial activity. Further Minimum Inhibitory Concentration analysis revealed that *Myrtus communis*, *Salvia officinalis*, and the essential oil blend inhibited *Staphylococcus xylosus* growth at concentrations between 0.5 and 0.25 percent, while *Cistus ladanifer* required higher levels (1 to 0.5 percent). These findings suggest that selected essential oils—especially in combination—hold promise as complementary antimicrobial agents in food safety and antimicrobial resistance mitigation efforts.

## Simple Summary

Milk can contain bacteria that are harmful to humans, especially when it comes from cows with early signs of udder infections that do not yet show symptoms. Some of these bacteria, like *Staphylococcus xylosus*, may be involved in the cheese-making process but can also pose risks to food safety. In this study, we found *Staphylococcus xylosus* in raw cow milk and tested whether certain plant-based essential oils could help control its growth. These oils came from three Mediterranean plants: myrtle, sage, and rockrose. We discovered that the oil blend had the ability to stop the bacteria from growing, especially when myrtle and sage were used. These results suggest that combining these essential oils could be a natural and effective way to improve the safety of milk and dairy products. This approach may also help reduce the use of traditional antibiotics, which is important in the fight against antimicrobial resistance. The findings could support safer food production and healthier outcomes for both animals and people.

## 1. Introduction

Bovine milk is not only a dietary staple but also a functional food with wide-ranging health implications [1], as emerging research highlighted its richness in bioactive peptides with potential health benefits, such as antioxidant function and antihypertensive [2].

Despite its nutritional benefits, it can serve as a reservoir for various pathogens if improperly handled or processed [3]. Among these, *Staphylococcus aureus* is a significant concern, as it can produce heat-stable enterotoxins that lead to food poisoning, even if the milk is subsequently pasteurized [4]. Other pathogens like *Salmonella* spp., *Listeria monocytogenes*, *Escherichia coli* (*E. coli*), and *Campylobacter* spp. are also common culprits in foodborne illnesses linked to milk consumption [3]. These risks are particularly pronounced in raw milk, where pasteurization—a process that destroys harmful microbes—is not applied [5]. Contamination can occur during milking due to poor hygiene, infected udders, or unsanitary equipment [6]. Addressing these risks requires stringent on-farm hygiene practices, routine microbial testing, and cold-chain maintenance during storage and transport [5]. Coagulase-negative staphylococci can lead to both clinical and subclinical mastitis, although subclinical infections are more common. On many dairy farms, subclinical mastitis can affect 40% to 80% of cows over the course of a year, and more than 15 different species of coagulase-negative staphylococci have been associated with mastitis cases, with *Staphylococcus xylosus* frequently identified as a dominant species in subclinical infections [7].

For this reason, cheesemaking with raw milk presents challenges, as harmful bacteria (e.g., the previously mentioned *S. xylosus*) can survive and sometimes proliferate [8]. Soft and semi-soft cheeses are especially vulnerable due to their high moisture and short ripening times, which promote pathogen growth [9]. Moreover, antimicrobial resistance (AMR) is a major global health threat affecting both public health and food safety. The dairy sector is particularly exposed, as microbial consortia influence product quality but may also harbor antibiotic-resistant bacteria [10,11] *Staphylococcus xylosus*, a common dairy commensal, contributes to fermentation but can act as an opportunistic pathogen [11,12]. Recent studies reveal AMR-associated proteins in milk bacteria, raising concerns about dairy products as potential reservoirs of resistant strains [1]. Innovative control strategies are needed to ensure microbiological safety without compromising quality or shelf life.

In this context, there is a growing focus on the use of alternative antimicrobial agents, with particular attention being given to essential oils. Essential oils are complex mixtures of natural compounds extracted from aromatic plants, renowned for their antibacterial, antifungal, and antiviral properties [13,14]. Their efficacy is attributed to the presence of alkaloids, flavonoids, terpenes, and phenolics, which act on various bacterial structures, disrupting membranes and inhibiting vital processes [15]. The use of essential oil blends is particularly promising, as synergistic interactions between the components can enhance their overall antimicrobial activity [16]. Phytobiotic combinations have already been demonstrated to be successful in chickens as an alternative to Antibiotic Growth Promoters (AGPs) when added to feed [17].

In the dairy sector, the application of essential oils could represent a natural and effective solution in the clinical field; for example, through topical use for the control of mastitis. This strategy would not only improve animal health management but also help reduce microbial loads in dairy products, thereby extending their shelf life and limiting the risk of contamination by resistant bacteria.

The first aim of this study is to identify possible bacterial pathogens that might be present in cows’ subclinical infections that might represent a threat to dairy products (especially the ones where raw milk is used). The second aim is to evaluate possible mitigating strategies to be applied in the field or during cheesemaking based on an essential oils blend (designed according to a previous study [15]) composed of *Myrtus communis*, *Salvia officinalis*, and *Cistus ladanifer*.

## 2. Materials and Methods

### 2.1. Sample Collection

In total, 14 samples were collected from non-primiparous Frisona (Holstein Friesian; mastitis IR 0.15 cow/month), Bruna Alpina, and Pezzata Rossa (mastitis IR 0.20 cow/month) cows from two different farms in Calabria (Italy). The samples were collected in two stages from 7 animals during preclinical (7 samples) and clinical (7 samples) mastitis, as reported in Supplementary Table S1. The preclinical samples were collected when mastitis condition was suspected according to the detection of small milk clots after stripping and visual inspection on a dark surface. Teats were pre-dipped for 20–30 s, disinfected with individual alcohol-soaked swabs until visibly clean, and dried with disposable towels before foremilk was stripped. Milk was collected into sterile pots held at a 45° angle, labeled, and stored separately. Collected samples were frozen (−80 °C) within 24 h [18].

### 2.2. Bacterial Isolation

The milk collected by all four quarters was pooled before seeding. For each sample, 100 µL of milk was then plated (in triplicate) on Mannitol Salt Agar (MSA) for Gram-positive bacterial isolation, and another 100 µL was plated on MacConkey agar for Gram-negative bacterial isolation. Following incubation (37 °C for 24 h), a single colony was picked for further analysis, e.g., susceptibility tests and bacterial species official identification.

Seven among the fourteen samples collected yielded a successful bacterial isolation in the preclinical or clinical sample. Among the successful isolations, three grew in the selective medium MacConkey agar, and the isolation was achieved only in the clinical samples. The remaining four isolates grew in the selective medium Mannitol salt agar, and only the pathogens (*Staphylococcus* spp.) capable of fermenting mannitol were retained for further analysis. Three were successfully isolated in the clinical samples, and only one in the subclinical sample. Overall, only in one case, the preclinical and clinical samples of one cow correctly identified as the same pathogen, posing the risk of bulk tank contamination.

### 2.3. Bacterial Identification

Bacterial strain identification was conducted using the MALDI Biotyper system (Bruker, Billerica, MA, USA), which relies on mass spectrometry for accurate spectral matching, ensuring precise organism identification. Briefly, a single bacterial colony was directly deposited onto an MTP BigAnchorChip 384 TF target plate (Bruker Daltonics, Billerica, MA, USA). The sample was then overlaid with 1 µL of α-CHCA matrix solution, a saturated mixture of alpha-cyano-4-hydroxycinnamic acid in 50% acetonitrile with 2.5% trifluoroacetic acid, and air-dried at room temperature to facilitate co-crystallization with the sample. Mass spectra were automatically imported into the BioTyper software (version 2.0, Bruker Daltonics, Billerica, MA, USA) for analysis via standard pattern matching. The process, from MALDI-TOF MS acquisition to identification, was fully automated. The software identified up to 100 peaks per spectrum, applying a signal-to-noise threshold of 10. Peaks with a mass-to-charge (*m*/*z*) difference of less than 250 ppm were considered identical after alignment. The resulting peak lists were matched against the reference database using the integrated pattern-matching algorithm. Identification scores were assigned as follows: scores below 1.7 were considered unreliable, scores between 1.7 and 1.99 indicated genus-level identification, and scores of 2.0 or higher confirmed species-level identification, in line with manufacturer guidelines. The raw spectral data were processed using MALDI BioTyper Automation 2.0 software (Bruker Daltonics).

### 2.4. Susceptibility Testing

For susceptibility testing, 100 µL of an *S. xylosus* suspension was spread-plated on MSA. The susceptibility was tested against the commercially available essential oils extracted from *Cistus ladanifer* (Aroma-Zone, Cabrières-d’Avignon, France), *Salvia officinalis* (Solimè srl, Cavriago, Italy), and *Myrtus communis* (Aroma-Zone, Cabrières-d’Avignon, France); the composition of each essential oil is reported in Table S3 in the Supplementary Materials. A blend of essential oils was prepared by combining equal parts of Myrtus communis, Salvia officinalis, and Cistus ladanifer in a 1:1:1 ratio. The resulting stock solution was then diluted to achieve final concentrations of 100 mg/mL, 50 mg/mL, and 25 mg/mL in ethanol. The antibacterial activity of the essential oil blend and erythromycin at different concentrations (6 µg/mL, 3 µg/mL, and 1.5 µg/mL) was assessed using the disk diffusion method (Whatman AA DISCS, Diameter 6 mm) in lawn-cultured plate count agar incubated at 37 °C for 24 h. The inhibition zones were measured using a digital caliper. The experiments were conducted in triplicate to ensure reproducibility, and the mean diameters with standard deviations (SD) were calculated for comparative analysis, as summarized in the tables.

### 2.5. Minimum Bactericidal Concentration (MBC) and Minimum Inhibitory Concentration (MIC) Evaluation

The bacterial viability (MBC) and growth (MIC) were both tested in the presence of the essential oils (singularly and mixed) in 96-well multi-plates. Briefly, the viability of bacterial cells was tested after 24 h exposure to the single essential oils or the blend at concentrations ranging from 16.60% to 0.01% in tryptic soy broth (TSB). Erythromycin was tested at concentrations ranging from 166 µg/mL to 0.1 µg/mL, and the survival of bacterial cells was tested by plating 10 µL of each well in MSA. The growth of bacterial cells was tested after 24 h exposure to the single essential oils or the blend at concentrations ranging from 16.60% to 0.01% in MSA and from 166 µg/mL to 0.1 µg/mL of erythromycin directly mixed in MSA. The inhibition/growth was confirmed by visually inspecting the wells of the plate with optical microscopy. No statistical analysis was needed as the three experimental replicates yielded the same results for MBC and MIC.

## 3. Results

### 3.1. Bacterial Pathogen Isolation and Identification

The isolation of bacterial samples from preclinical and mastitic milk was performed in mannitol salt agar, which is the selective culture medium for halophilic bacteria. In both isolated samples, the culture medium turned yellow after the bacterial growth, and it was possible to hypothesize the presence of a *Staphylococcus* spp. able to ferment mannitol. The hypothesized results were confirmed by MALDI Biotyper analysis, which, as shown in Table 1, Supplementary Table S1, and Supplementary Figure S1, demonstrated with good confidence that preclinical and clinical samples carried vital forms of *Staphylococcus xylosus.* All analyzed samples yielded high-confidence scores (ranging from 1.87 to 2.19) and consistently identified *S. xylosus* (a representative spectrum-*m*/*z* of 2000 to 20,000 obtained from the analysis is shown in Supplementary Figure S1). Going deeper into the identification, the most probable identification can be assigned to the strain DSM 20266T, and better identification scores were obtained with the second isolation (Table S2). The identification results (triplicate) relative to the first isolation are represented in the first two rows (1a and 1c) of Table 1 and  Table S2, while the last three rows (2a, 2b, and 2c) shown are the identifications relative to the second isolation. As the two different isolates were independently sampled 4 days apart, respectively, during preclinical and clinical mastitis, it is relevant to highlight the confidence obtained with the same identification and the fact that better identification scores were obtained for the second sampling.

### 3.2. Susceptibility Testing

After the bacterial isolation and identification, the plates were seeded with a lawn of *Staphylococcus xylosus* to be subsequently tested with the selected essential oils and with the erythromycin antibiotic. The essential oils blend was designed according to the findings previously described [15,19], reporting the most effective plant extracts being *Salvia officinalis*, *Cistus monspeliensis*, *Cistus salviifolius*, *Origanum vulgare*, and *Myrtus communis,* with recorded average MICs against *S. aures* of 0.46 mg/mL, 0.72 mg/mL, 0.83 mg/mL, 1.02, and 0.74, respectively. The three extracts with the lowest annotated MIC were chosen, purchased, and included in this study.

**Figure 1:**
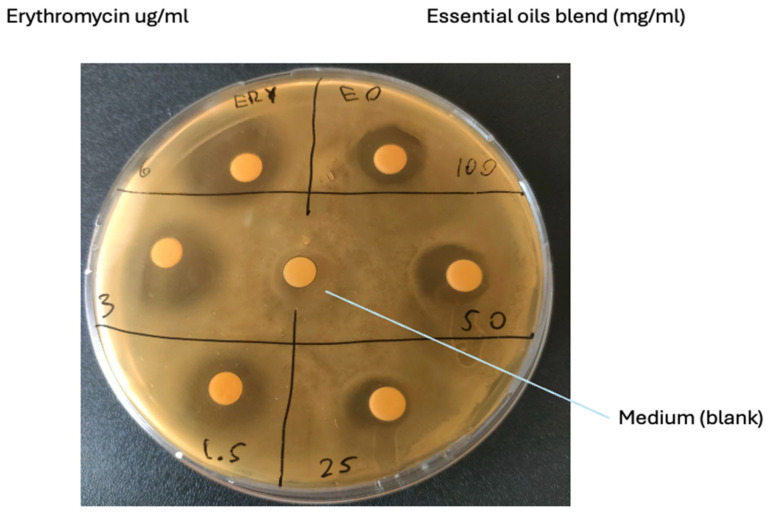
Representative image of erythromycin and essential oil blend inhibition zones at various concentrations obtained through the Kirby–Bauer test. The numbers in the quadrants relative to the essential oils (right side) represent the concentration expressed in mg/mL, and the numbers on the left side relative to erythromycin represent the concentration expressed in µg/mL.

The inhibitory effects of the essential oil blend and erythromycin on *S. xylosus* are summarized in Table 2. Erythromycin demonstrated dose-dependent inhibition, with the largest average zone diameter observed at the highest concentration (6 µg/mL) (Figure 1). The essential oils blend exhibited inhibition zones ranging from 9 mm to 13.33 mm, depending on the concentration.

### 3.3. MBC and MIC Evaluation

**Figure 2:**
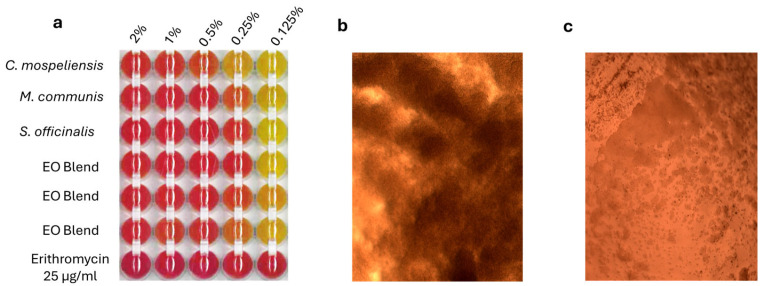
(**a**) Multi-well plate detail showing the bacterial inhibition (red) and growth (yellow) in MSA. (**b**) Microscope (10× objective) picture of the well where bacterial growth was present (yellow). (**c**) Microscope picture (10× objective) of the well where bacterial growth was absent (red).

Considering the preliminary results obtained from the sensitivity testing, it was decided to further progress with MBC and MIC (Figure 2) evaluation. Regarding MBC, no essential oil, alone or in combination with the others, showed bactericidal activity. On the other hand, erythromycin showed an MBC between 0.6 µg/mL and 0.36 µg/mL.

Table 3 shows the Minimum Inhibitory Concentration (MIC) percentages for each essential oil against *S. xylosus*. *Cistus ladanifer* exhibited a MIC range of 1–0.5%, while *Myrtus communis*, *Salvia officinalis*, and the essential oil blend all showed an MIC range of 0.5–0.25%, demonstrating a similar antimicrobial potency. Erythromycin, used as a positive control, had a significantly lower MIC (<0.1 µg/mL).

## 4. Discussion

*S. xylosus* presence in milk during subclinical mastitis could persist through cheese production and represent a source of food safety risks for dairy products made with unpasteurized milk [20]. Using a meta-analytical and a bench-to-practice translational approach, we designed this essential oil blend that represents an eco-friendly strategy to reduce AMR spread via dairy products. Its application may serve dual purposes: pathogen control and natural preservation during cheese fermentation. The blend’s antimicrobial activity stems from membrane disruption, enzymatic inhibition, and metabolic interference. This research builds on previously published meta-analyses identifying *Myrtus communis*, *Salvia officinalis*, *and Cistus ladanifer* as promising antimicrobial candidates [15]. The innovation lies in using a blend of these botanicals—traditionally active against *S. aureus*—to target *S. xylosus*, a less-studied but increasingly relevant mastitis pathogen. Unlike conventional antibiotics, the essential oil blend exhibits multi-target antimicrobial activity, reducing the risk of resistance development [21,22].

Coagulase-negative staphylococci are widespread in nature and are known to inhabit the skin and mucosal surfaces of humans and animals. Once thought to be harmless, they have now been recognized as a growing cause of bovine mastitis across various regions worldwide [23,24,25]. Subclinical mastitis in cows silently affects herd health due to its asymptomatic nature, often evading detection and intervention by farmers, and often causing acute clinical episodes [26]. Over 15 different species of coagulase-negative staphylococci have been recognized as contributors to the onset of mastitis [27], and among those [25,28], *S. xylosus* emerged as the most predominant isolate.

Considering this premise about their subclinical presence, there is the concrete possibility that these identified bacterial species/strains can easily step further into the food chain through dairy products. Lactic acid bacteria are particularly important for cheese making [29]; however, also *staphylococci* represent key components of the bacterial core in cheese and were detected in various cheese types [30,31]. Furthermore, *S. xylosus* is commonly found in animal-derived food products and is frequently utilized as a starter culture due to its role in enhancing flavor. However, it has the ability to form biofilm, and its potential pathogenic impact should not be overlooked [32]. For example, dairy mastitis caused by *Staphylococcus xylosus* has become a significant issue in the dairy industry, and this bacterium was consistently isolated from the cheeses growing to 10^5^–10^9^ cfu/g, depending on the product [33]. This is representative of the fact that this bacterium can easily jump from the collected milk, especially in cases of its subclinical presence in the herd, to the cheese, and that it can persist during the fermentation/maturation processes of dairy products.

As shown in Table 1, its identification was consistent in preclinical and clinical mastitis. This isolate exhibited sensitivity to erythromycin in a dose-dependent manner, starting from 1.5 µg/mL, not posing the problem of a possible AMR to macrolides. However, other *S. xylosus* isolates from bovine mastitis have been reported to carry the erm (44) gene [34], which codes for a methylase that confers resistance to macrolide, lincosamide, and streptogramin B antibiotics. The global increase in antimicrobial resistance (AMR) poses a significant challenge to public health, necessitating the search for alternative therapeutic approaches. Among these alternatives, plant extracts, particularly essential oils, have gained attention for their broad-spectrum antimicrobial properties. Moreover, the multi-target action of phytocomplexes, rather than the single molecule, makes it more difficult for the bacteria to develop resistance mechanisms. In this context, we chose to use the extracts of the plants that, according to our previously published study [15], had the highest chance of being effective against *S. aureus*, and we decided to measure their possible activity against the isolate detected herein, *S. xylosus*.

The essential oil blend composed of *Myrtus communis*, *Salvia officinalis*, and *Cistus ladanifer* demonstrated a dose-dependent inhibitory effect of the essential oil blend, highlighting its potential as a complementary antimicrobial agent. The disk diffusion method used to assess the essential oil blend’s efficacy showed (Table 3 and Figure 1) inhibition zones ranging from 13.3 mm at the highest concentration (100 mg/mL) to 9 mm at the lowest concentration (25 mg/mL), demonstrating its antimicrobial potential. This result aligns with previous studies on essential oils, particularly those derived from *Myrtus communis* and *Salvia officinalis*, which are known for their antimicrobial properties due to phenolic compounds, terpenes, and flavonoids. None of the essential oils, whether used individually or in combination, exhibited bactericidal activity, as confirmed by the absence of MBC within the tested concentration range. In contrast, erythromycin demonstrated a clear bactericidal effect, with MBC values ranging from 0.36 to 0.6 µg/mL, aligning with its established efficacy against Gram-positive bacteria.

Regarding MIC values, *Myrtus communis*, *Salvia officinalis*, and the essential oil blend all showed comparable inhibitory activity, with MICs between 0.5% and 0.25%, indicating consistent antimicrobial properties. *Cistus ladanifer* was less effective, requiring higher concentrations (1–0.5%) to achieve inhibition. The antimicrobial activity of essential oils is primarily linked to their ability to disrupt bacterial cell membranes, interfere with enzymatic functions, and inhibit essential metabolic pathways [35]. The multi-target nature of essential oils reduces the likelihood of bacteria developing resistance, making them a valuable tool in the fight against AMR. In the context of dairy production, where *S. xylosus* plays both beneficial and potentially harmful roles, the use of essential oils could offer a dual benefit. They could help maintain microbial balance by selectively inhibiting pathogenic strains and serve as natural preservatives to enhance food safety and shelf life.

## 5. Conclusions

This study highlights the detection of *Staphylococcus xylosus* in both preclinical and clinical bovine mastitis, underlining its potential to silently enter the dairy production chain, especially in raw milk cheesemaking. Given its dual role as both a traditional flavor-enhancing microorganism and an emerging mastitis-associated pathogen with biofilm-forming ability, *S. xylosus* poses a food safety risk when present in unpasteurized dairy products. In this case, we adopted a blend of essential oils extracted from Mediterranean plants, which was found to be effective against this microorganism and might be beneficial as a topical treatment for preclinical mastitis or as a co-adjuvant to limit its growth in further steps of dairy product processing.

## Figures and Tables

**Table 1:** MALDI Biotyper identification results (species level).

Sample ID	Best Match	Score Value	Second-Best Match	Score Value
cow 1a	*Staphylococcus xylosus*	2.13	*Staphylococcus xylosus*	2.05
cow 1c	*Staphylococcus xylosus*	1.87	*Staphylococcus xylosus*	1.81
cow 2a	*Staphylococcus xylosus*	2.05	*Staphylococcus xylosus*	1.98
cow 2b	*Staphylococcus xylosus*	2.19	*Staphylococcus xylosus*	2.19
cow 2c	*Staphylococcus xylosus*	2.07	*Staphylococcus xylosus*	1.83

**Table 2:** Measured inhibition zones for erythromycin and EO blend.

Treatment	Average Inhibition Zone (Diameter; mm-SD)	Zone Diameter Interpretative Standards for *Staphylococcus* Species (mm) *	Antibiotic Susceptibility Results
Ethanol (medium)	6-0		No inhibition
EO Blend 100 mg/mL	13.33-1.15		Sensible
EO Blend 50 mg/mL	15.33-9.40		Sensible
EO Blend 25 mg/mL	9-1		Sensible
Erythromycin 6 µg/mL	23-4.59	≥5.2	Sensible
Erythromycin 3 µg/mL	18.6-3.51	≥2.6	Sensible
Erythromycin 1.5 µg/mL	15.33-3.05	≥1.3	Sensible

* The results of antibiotic susceptibility testing for erythromycin were interpreted based on the reference values provided by the CLSI (Clinical and Laboratory Standards Institute). The reference values were normalized to the concentrations used in our study, with the CLSI indicating a reference concentration of 15 micrograms.

**Table 3:** Table showing the calculated MICs of the single essential oils, the essential oils blend, and erythromycin.

Treatment	Minimum Inhibitory Concentration %
*Cistus ladanifer*	1–0.5
*Mirtus communis*	0.5–0.25
*Salvia officinalis*	0.5–0.25
essential oil blend	0.5–0.25
Erythromycin	<0.1 µg/mL

## Data Availability

All necessary data are provided within this document and with the Supplementary Information.

## Supplementary Materials

The following supporting information can be downloaded at: https://www.mdpi.com/article/10.3390/vetsci12070659/s1, Figure S1: Representative spectrum of *S. xylosus* identified from cow’s milk; Table S1: Milk samples collected for bacterial isolation and study; Table S2: MALDI Biotyper Identification of the Bacterial Strain Results; Table S3: Composition of used EOs.
