# Supplementary material for: Mitigating Bovine Mastitis and Raw Milk Pathogen Risks: Inhibition of Staphylococcus xylosus by Mediterranean Plants’ Essential Oil
Source: Vet Sci. 2025 Jul 11;12(7):659. doi: 10.3390/vetsci12070659 (PMC12299493; doi:10.3390/vetsci12070659)
Supplement: Supplementary file 1 [file vetsci-12-00659-s001.zip › vetsci-3672989-supplementary.pdf]

Supplementary material submitted along with the manuscript:

Mitigating Bovine Mastitis and Raw Milk Pathogen Risks: Inhibition of *Staphylococcus xylosus* by Mediterranean Plants' Essential Oil

Rosario De Fazio [1], Giacomo Di Giacinto [2], Paola Roncada [1], Domenico Britti [1,3], Rosangela Odore [2], Paola Badino [2,†] and Cristian Piras [1,3,*,†]

[1] Department of Health Sciences, Magna Græcia University of Catanzaro, 88100 Catanzaro, Italy; rosario.defazio@studenti.unicz.it (R.D.F.); roncada@unicz.it (P.R.); britti@unicz.it (D.B.)
[2] Department of Veterinary Sciences, University of Turin, Largo Braccini 2, Grugliasco, 10095 Torino, Italy; giacomo.digiacinto@unito.it (G.D.G.); rosangela.odore@unito.it (R.O.); paola.badino@unito.it (P.B.)
[3] Interdepartmental Center Veterinary Service for Human and Animal Health, University "Magna Graecia" of Catanzaro, CISVetSUA, 88100 Catanzaro, Italy
* Correspondence: c.piras@unicz.it
† These authors contributed equally to this work.

Table S1. Milk samples collected for bacterial isolation and study.

Breed/animal code	Culture medium				SCC/days before the clinical diagnosis
	MacConkey agar		Mannitol salt agar (with mannitol fermentation)		
	Preclinical Mastitis	Clinical Mastitis	Preclinical Mastitis	Clinical Mastitis	
Frisona/F1	-	-	-	-	190000/18
Pezzata rossa/P1	-	+	-	-	450000/4
Frisona/F2	-	-	+	+	560000/4
Frisona/F3	-	-	-	+	300000/7
Bruna alpina/B1	-	-	-	+	720000/2
Frisona/F4	-	+	-	-	440000/8
Frisona/F5	-	+	-	-	290000/15

Table S2. MALDI Biotyper Identification of the Bacterial Strain Results.

Sample ID	Best Match	Score Value
cow 1a	*Staphylococcus xylosus DSM 20266T DSM*	2.13
cow 1c	*Staphylococcus xylosus FI FLR*	1.69
cow 2a	*Staphylococcus xylosus DSM 20266T DSM*	2.05
cow 2b	*Staphylococcus xylosus DSM 20266T DSM*	2.19
cow 2c	*Staphylococcus xylosus DSM 20266T DSM*	2.07

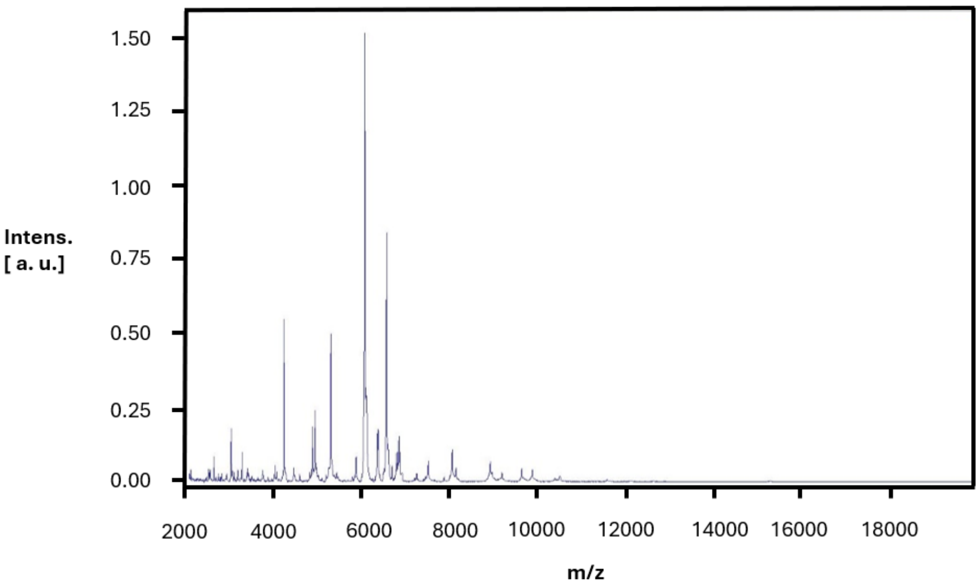

Figure S1. Representative spectrum of *S. xylosus* identified from cow's milk.

Table S3. Composition of used EOs.

**Scientific name/density**	**Composition**
Cistus ladanifer/0.903	42.65% Alpha-pinene; 5.33% Camphene; 2.02% Trans-pinocarveol; 3.57% Viridiflorol; 1.34% Borneol; 1.59% Trimethylcyclohexanone; 3.19% Acetate de bornyle
Myrtus communis/0.91	14.4% Acetate myrtenyle; 30.12% 1,8-cinéole; 25.67% Alpha-pinene; 11.76% Limonene; 2.34% Acetate geranyle; 4.04% Linalol; 0.94% Methylchavicol (estragol)
Salvia officinalis/0.906	35% Alpha thujone; 25% Canfora; 15% 1,8 cineole, 10% Alpha pinene; 10% Camphene; 5% Beta-caryophyllene; 5% Limonene.
